# Supplementary material for: Arginine methylation catalyzed by PRMT1 is required for B cell activation and differentiation
Source: Nat Commun. 2017 Oct 12;8:891. doi: 10.1038/s41467-017-01009-1 (PMC5638811; doi:10.1038/s41467-017-01009-1)
Supplement: Supplementary file 1 — Supplementary Information [file 41467_2017_1009_MOESM1_ESM.pdf]

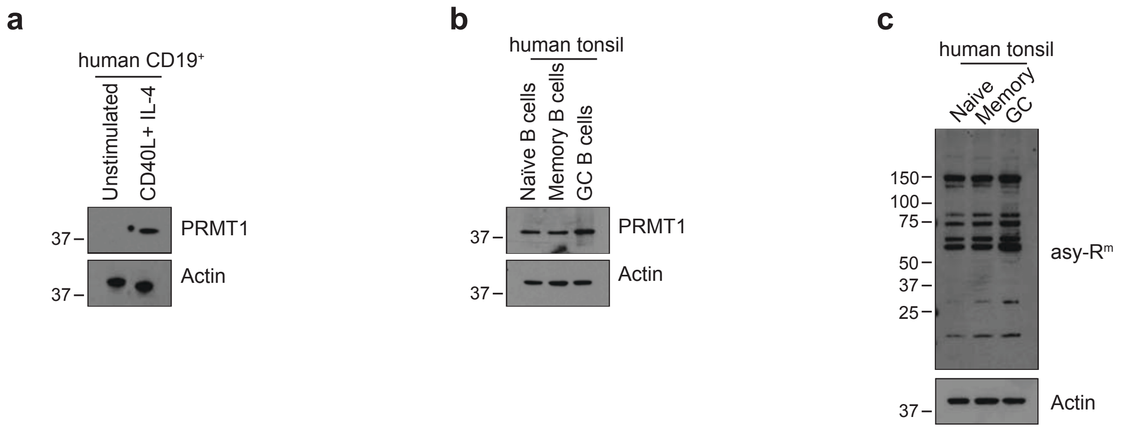

Supplementary Figure 1: Expression of PRMT1 in activated human B cells. (**a**) $CD19^+$ human B cells, isolated from PBMC of a healthy donor, were activated for three days with CD40L + IL4. Whole-cell lysates were then analyzed with a specific antibody for PRMT1 expression. (**b, c**) Naïve ($CD20^+CD27^-CD38^{lo}$), memory ($CD20^+CD27^+CD38^{lo}$) and GC ($CD20^{hi}CD38^{hi}CD27^+$) B cells were sorted from human tonsils, lysates prepared and probed for PRMT1 (**b**) and asymmetrically dimethylated arginine-containing proteins (**c**). (**a-c**) Equal loading was shown with anti-actin antibody. Entire Western blots are presented in Supplementary Fig. 5.

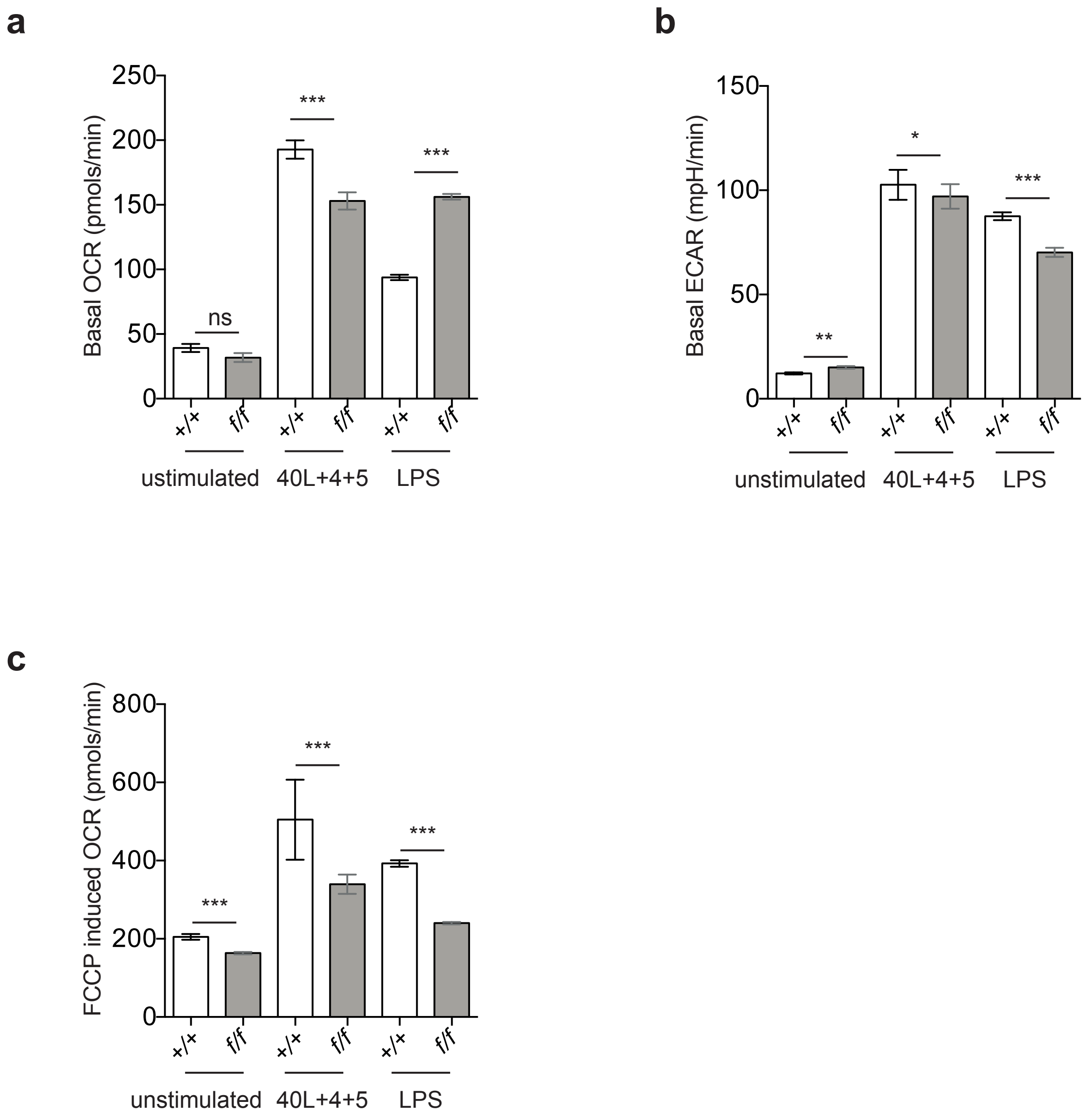

Supplementary Figure 2: Metabolic consequences of *Prmt1* deletion in B cells. (a-c) Oxygen-consumption rate (OCR) and glycolytic capacity measured by extracellular acidification rate (ECAR) was determined in naïve and activated B cells. (**a**) Basal OCR, (**b**) basal ECAR and (**c**) maximal respiratory capacity were determined by treatment with the electron transport chain-uncoupler FCCP (FCCP induced OCR) levels are indicated. Naïve and activated B cells were obtained from spleens of control (+/+) and *Prmt1*$^{f/f}$CD23Cre (*f/f*) mice. B cells were activated for 30hr *in vitro* with the indicated stimuli prior to measurements. * p ≤ 0.05, ** p ≤ 0.01, *** p ≤ 0.001, ns (not significant; unpaired t test). Data are representative of two independent experiments with 4 biological replicates. Mean and s.e.m in a, **b** and **c**.

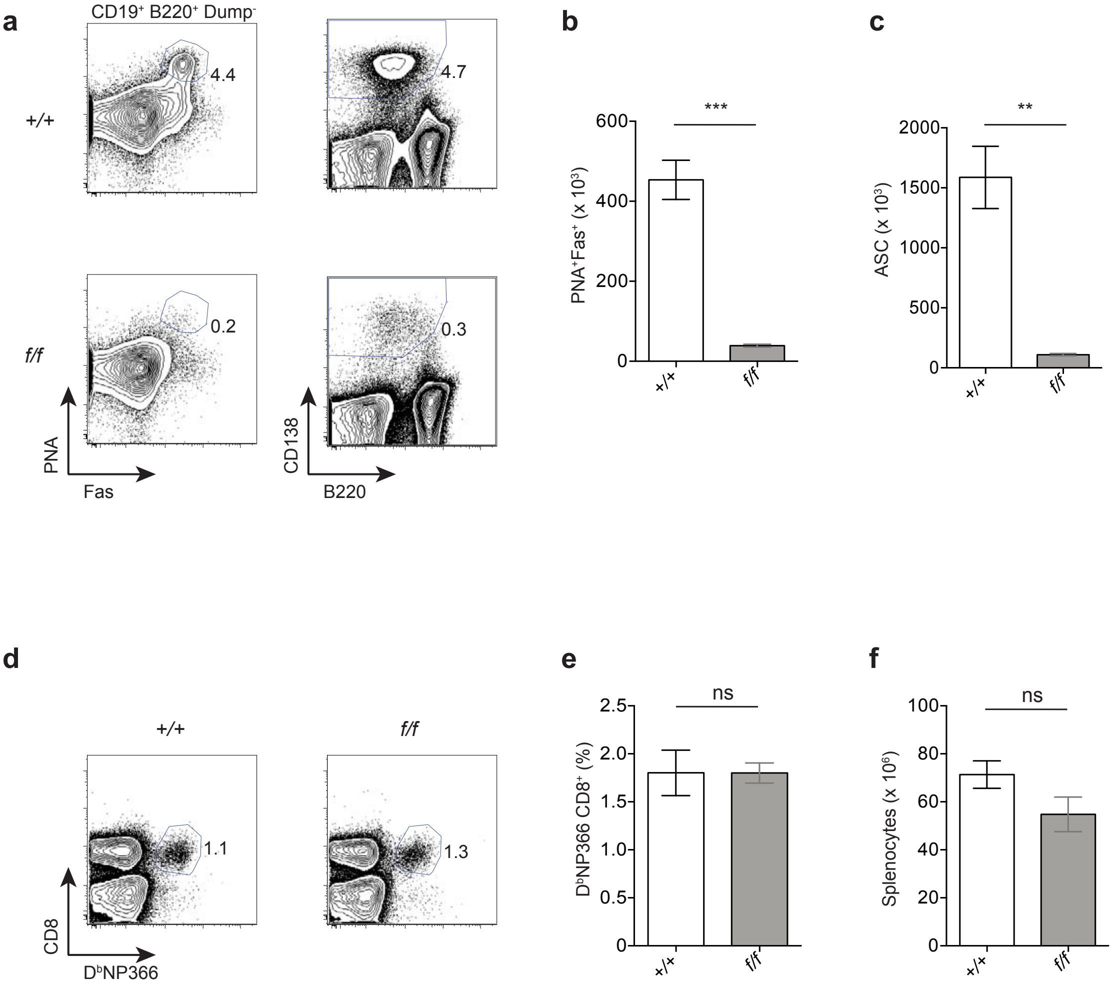

Supplementary Figure 3. *Prmt1*$^{f/f}$*CD23Cre* mice have an impaired immune response to influenza virus infection. (**a-f**) Control (*+/+*) and *Prmt1*$^{f/f}$*CD23Cre* (*f/f*) mice were infected intranasally with HKx31 influenza virus. (**a**) Flow cytometric analysis of splenocytes 7 days after infection. Representative dot plots show frequency of (left) GC B cells ($CD19^{+}B220^{+}Dump^{-}PNA^{+}Fas^{+}$) and (right) ASC ($CD138^{+}B220^{low}$). Numbers indicate percentage of the displayed events within the gated regions. (**b, c**) Graphs depict absolute number of spleen GC B cells (**b**), and ASC (**c**) at day 7 post-influenza virus infection for indicated strains. (**d-f**) Representative flow cytometric plot of DbNP366-specific splenic $CD8^{+}$ T cells, (**e**) percentage of DbNP366-specific $CD8^{+}$ T cells and (**f**) total splenocyte number are indicated for (*+/+*) and (*f/f*) infected mice. ** $p \leq 0.01$, *** $p \leq 0.001$ and ns (not significant; unpaired t test). The experiment comprises at least 5 mice per group. Mean and s.e.m. (**b, c, e, f**).

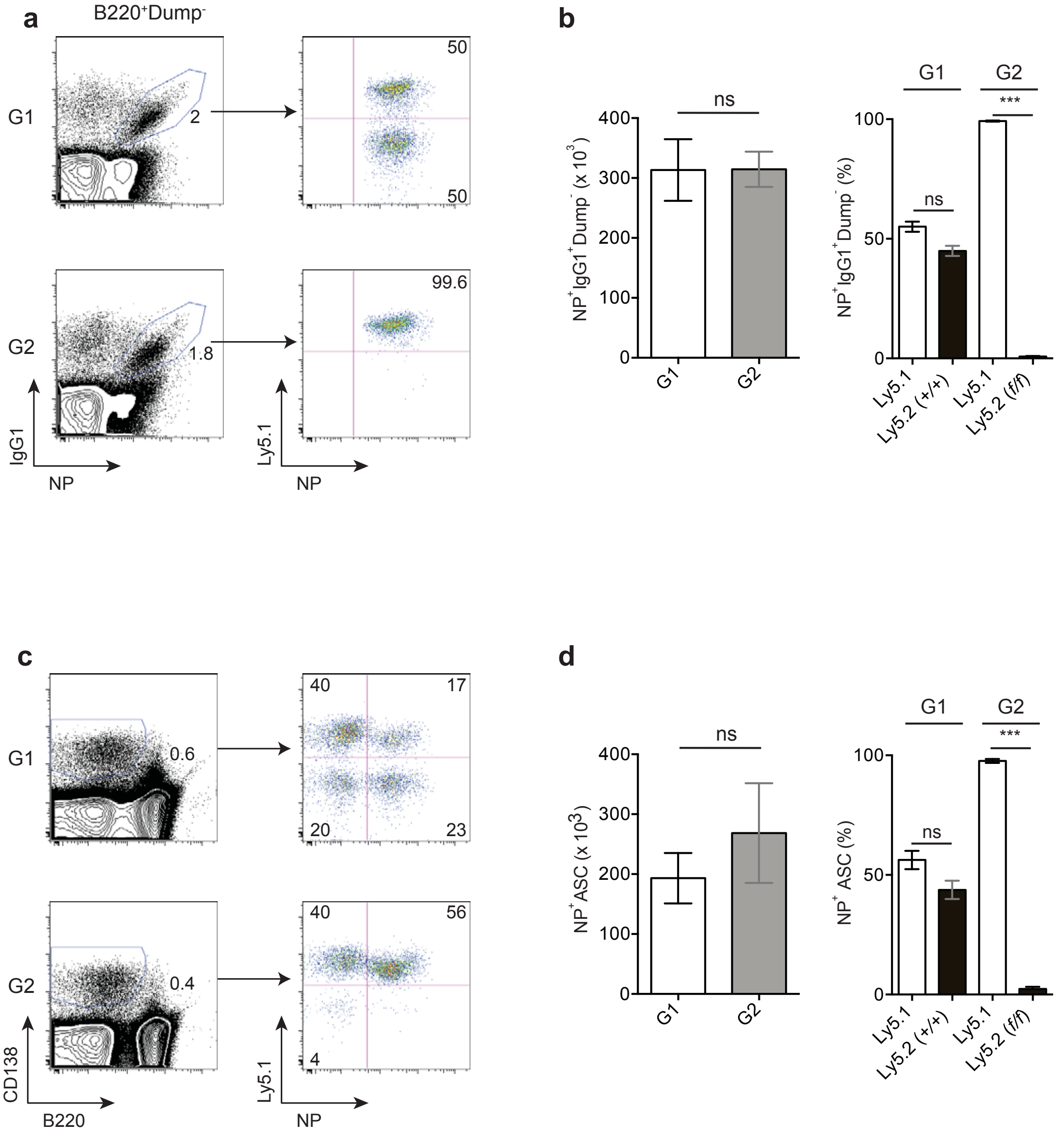

Supplementary Figure 4. PRMT1 is intrinsically required for antigen specific B cell expansion. (**a-d**) Irradiation chimeras were generated with (G1) 50% Ly5.1 and 50% CD23Cre (Ly5.2, *+/+*) or (G2) 50% Ly5.1 and 50% *Prmt1*$^{f/f}$*CD23Cre* (Ly5.2, *f/f*) BM cells. Nine weeks post reconstitution, mice were immunized with NP-KLH in alum. (**a, c**) Representative FACS plots of spleens 7 days post NP-KLH immunization revealing proportions of (**a**) antigen-reactive B cells and (**c**) total plasma cells with their allotype composition and NP-binding shown in the adjacent panel. BM donor groups are as indicated and numbers represent percentages of gated events. (**b, d**) Total number of NP-reactive IgG1$^+$ Dump$^-$ (IgM$^-$ IgD$^-$ Gr1$^-$) B cells (**b**) and NP$^+$ ASC (**d**) found in the spleen at day 7 after immunization in the indicated reconstitution groups. In each group, proportions are subdivided per genotype (Ly5.1$^+$ and Ly5.2$^+$) of the donor cells as indicated. *** $p \leq 0.001$, ns (not significant; unpaired t test). Data are representative of 6 mice per group. Mean and s.e.m in **b** and **d**. Flow cytometry gating strategies for this figure are shown in Supplementary Fig. 12.

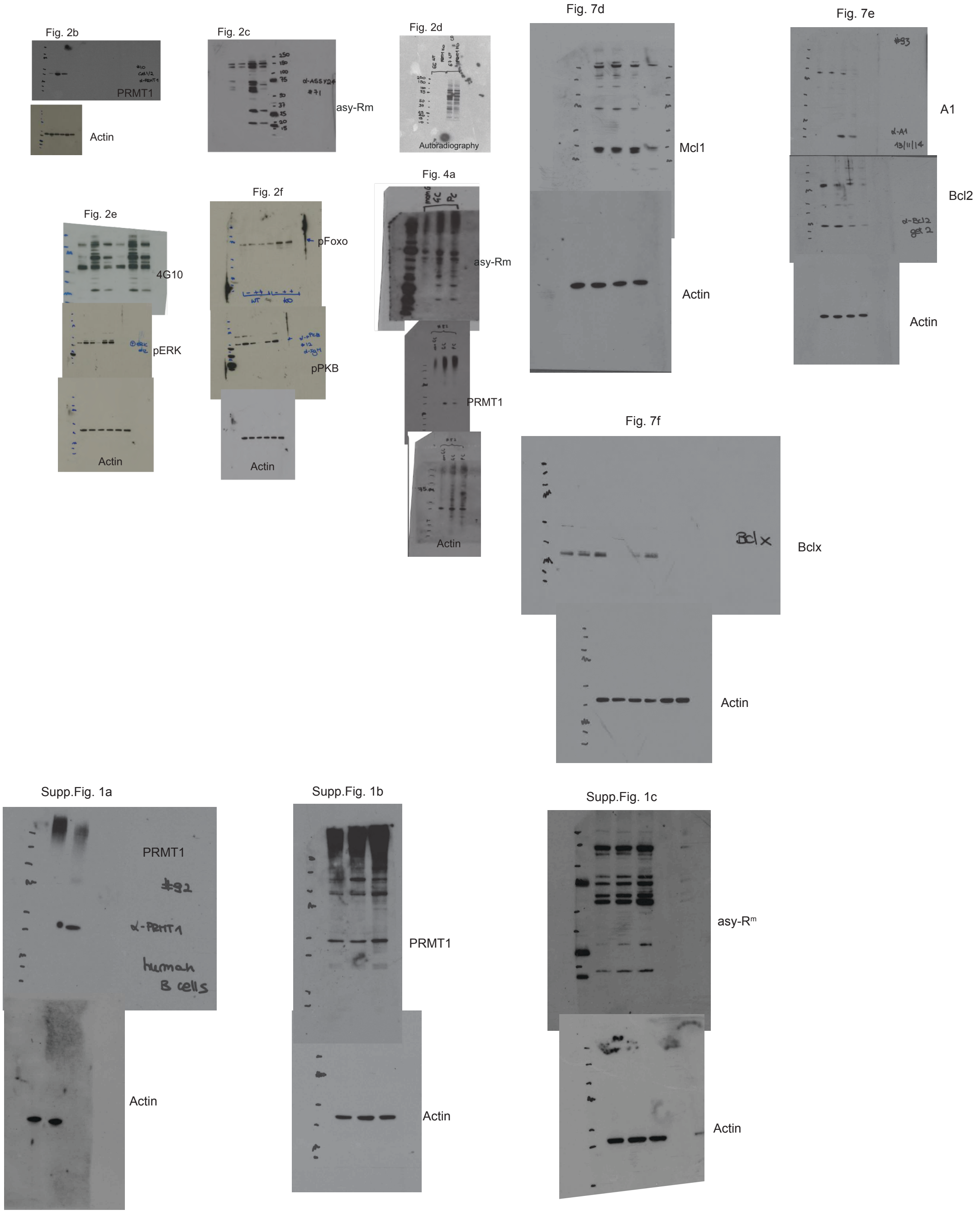

Supplementary Figure 5. Original exposures of Western blots and autoradiographs from the indicated figures and supplementary figures, probed for the indicated proteins or modifications.

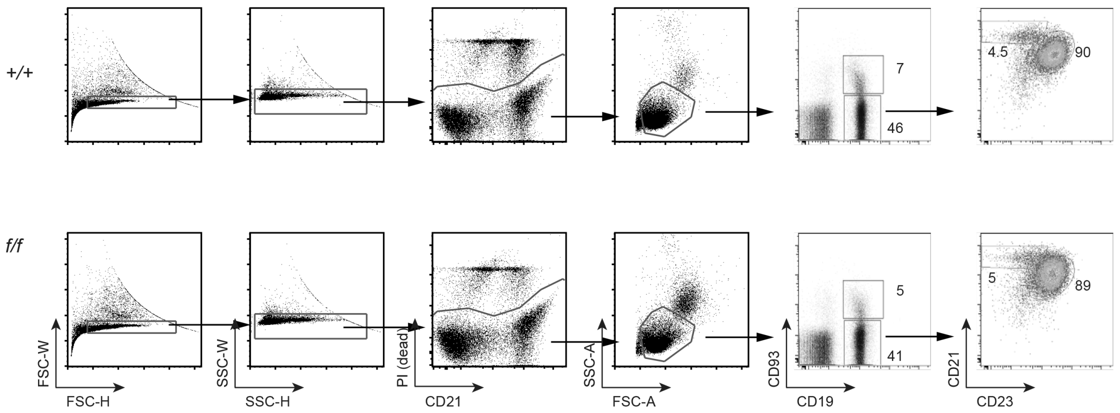

Supplementary Figure 6. Gating strategy for flow cytometry plots of Figure 1a.

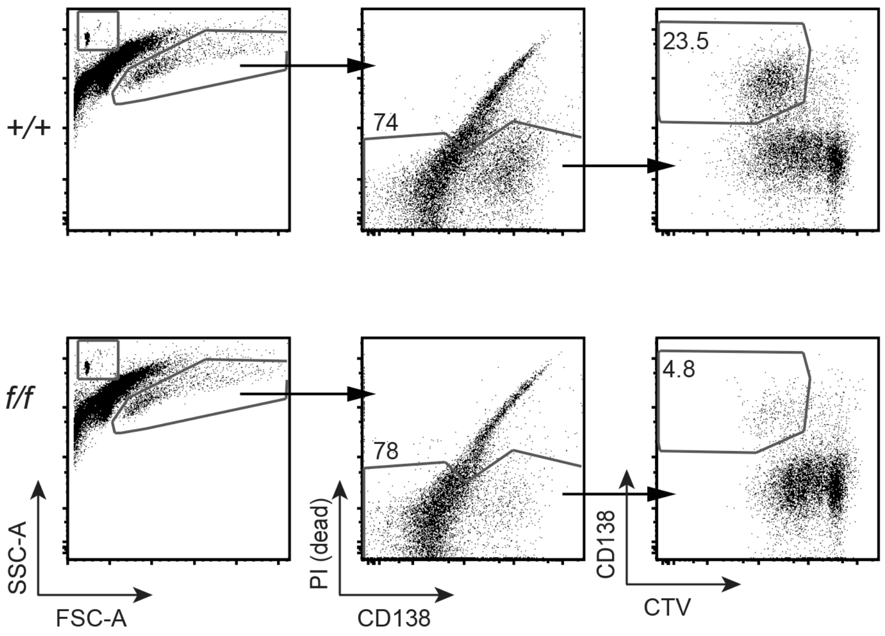

Supplementary Figure 7. Gating strategy for flow cytometry plots of Figure 3a.

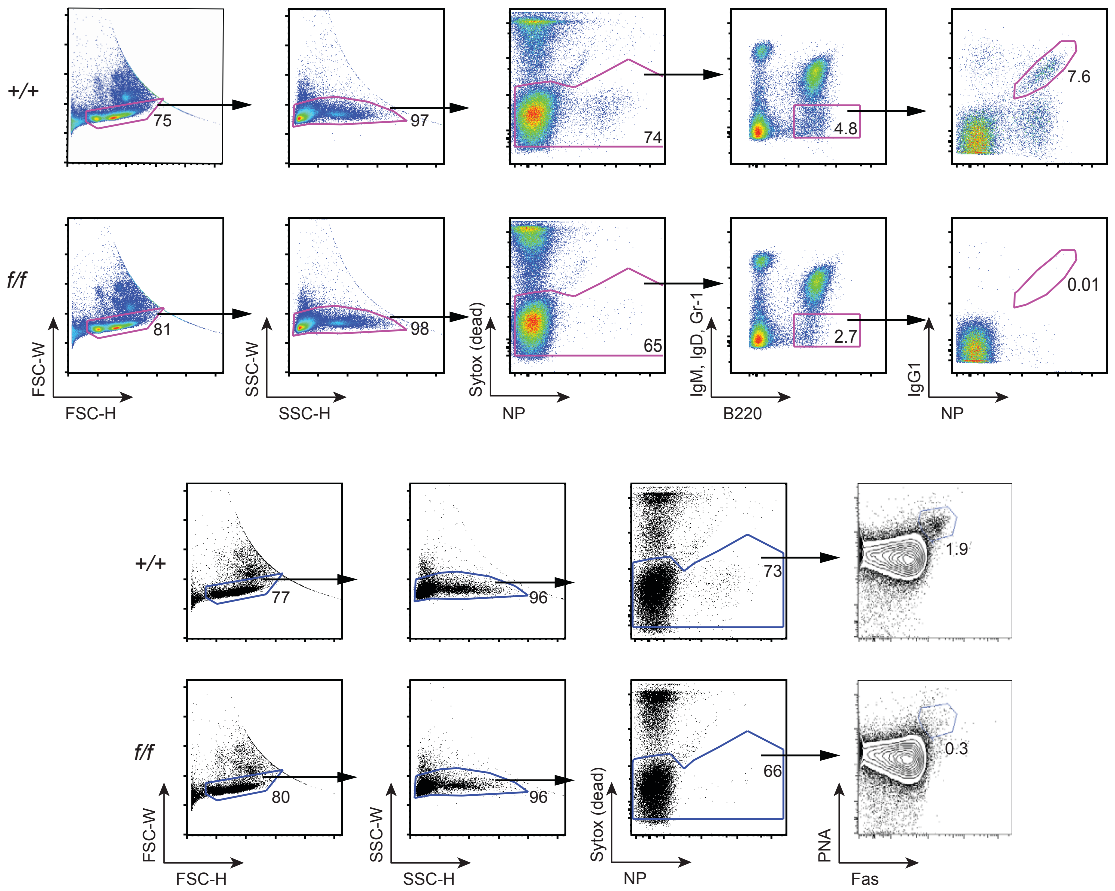

Supplementary Figure 8. Gating strategy for flow cytometry plots of Figure 4b NP staining (upper) and total GC staining (lower).

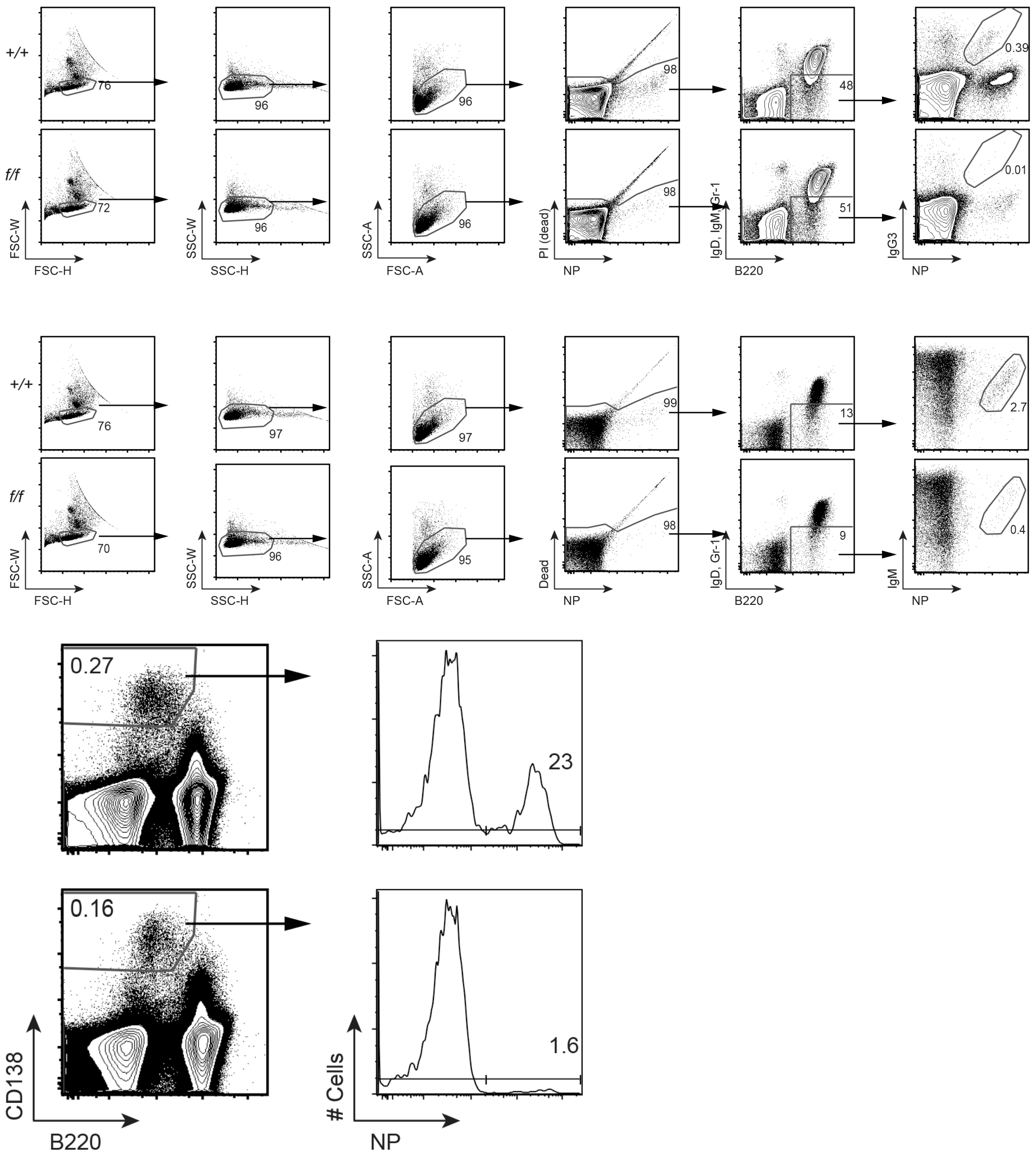

Supplementary Figure 9. Gating strategy for flow cytometry plots in Figure 5a (upper), 5b (center) and 5c (lower).

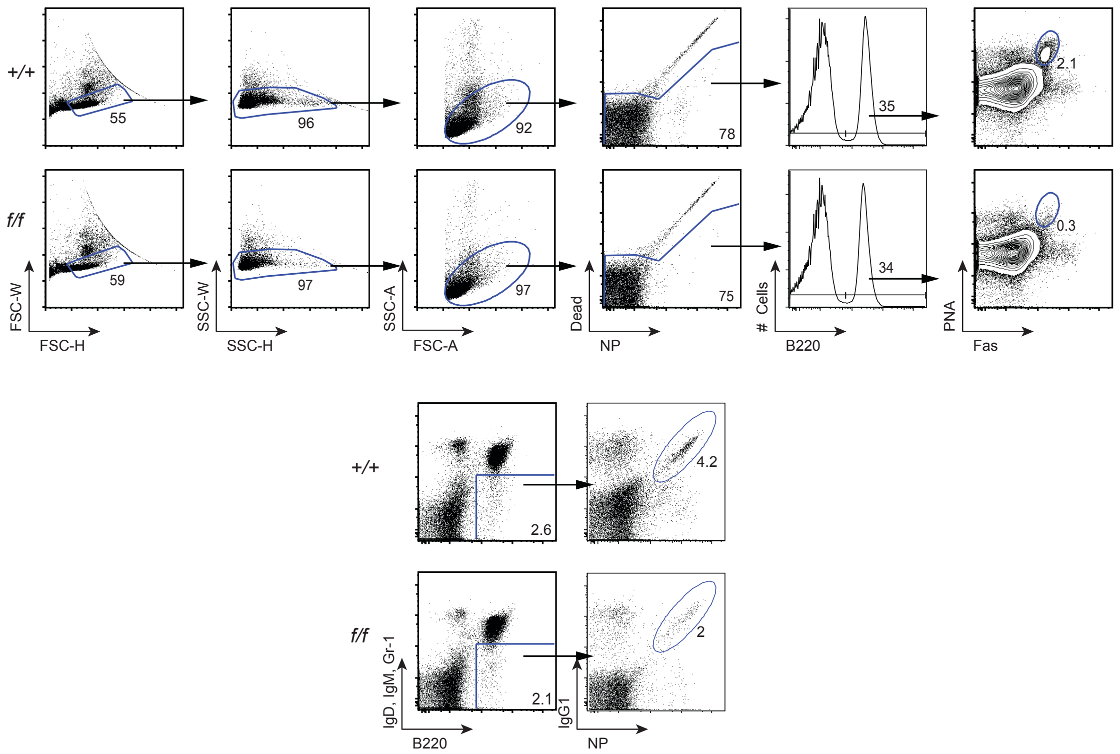

Supplementary Figure 10. Gating strategy for flow cytometry plots in Figure 6.

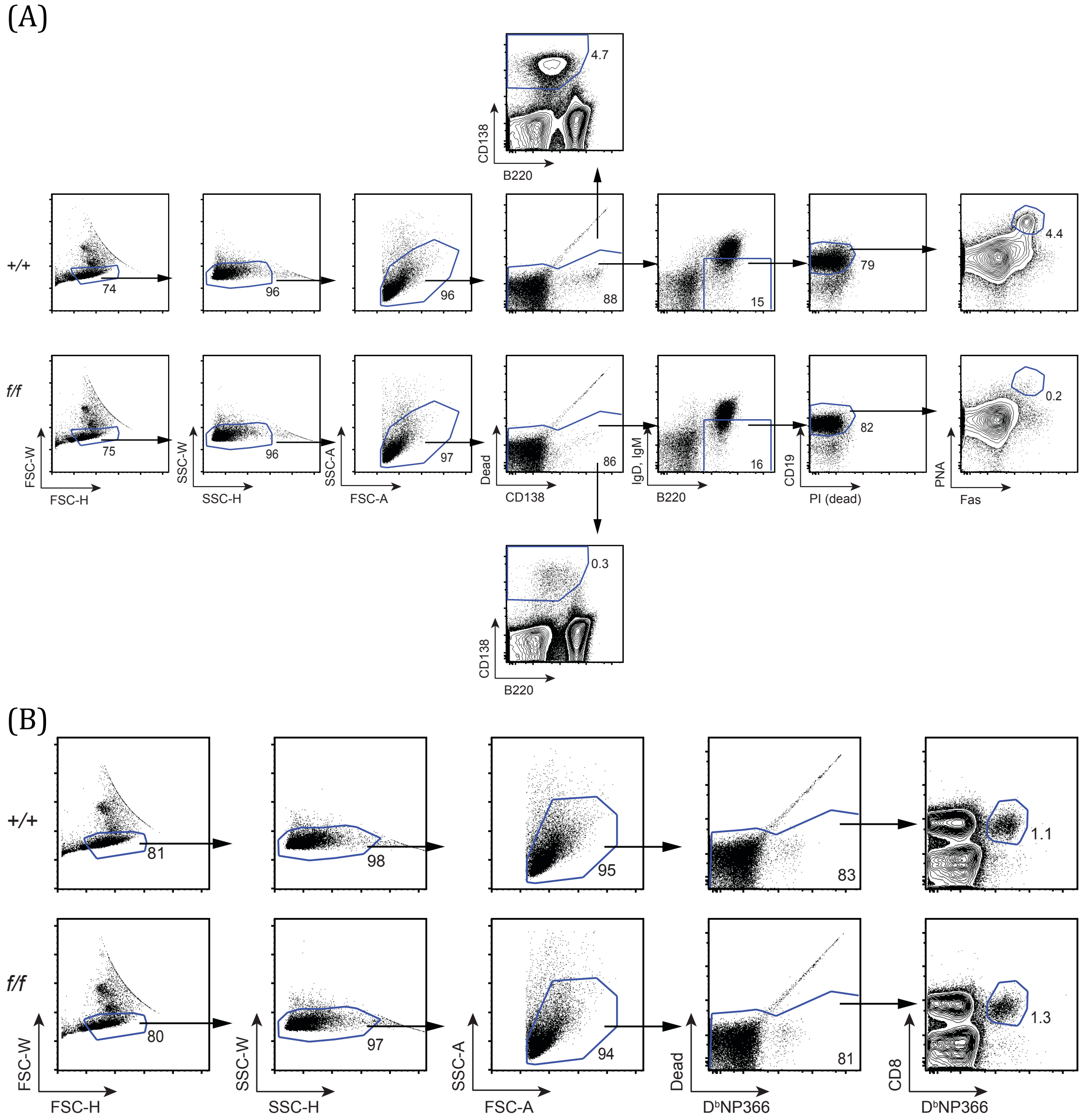

Supplementary Figure 11. Gating strategy for flow cytometry plots in (A) Supplementary Figure 3a and (B) Supplementary Figure 3d.

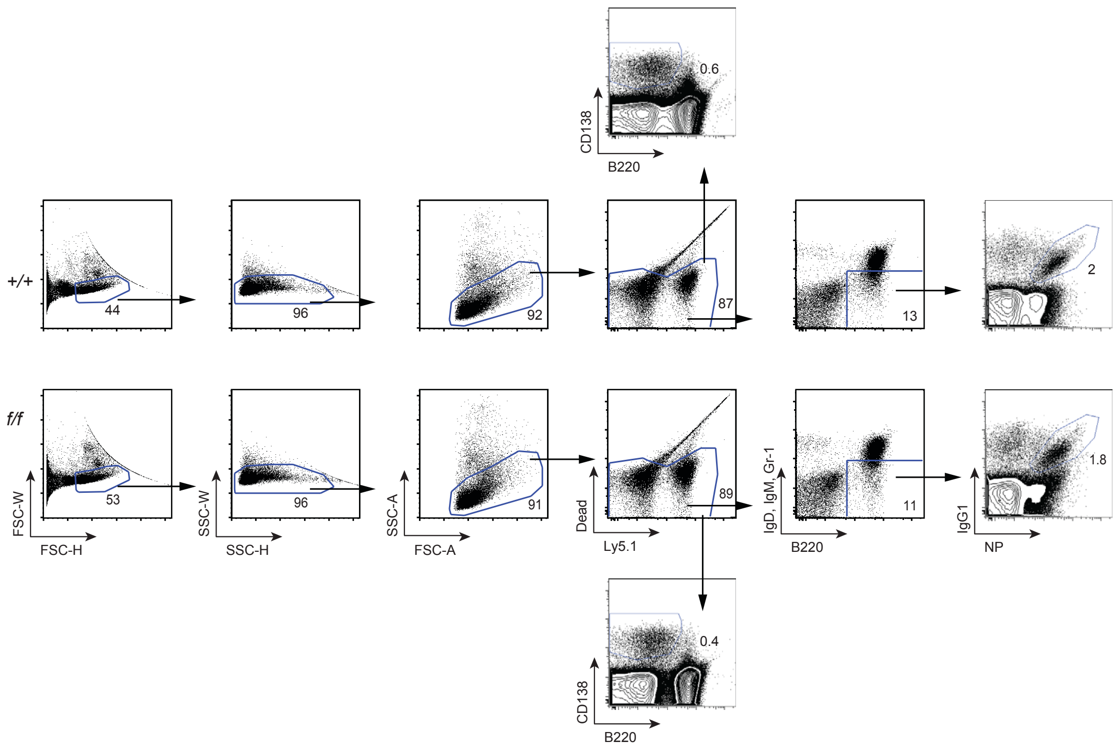

Supplementary Figure 12. Gating strategy for flow cytometry plots in Supplementary Figure 4.
